# Giant Gastric Trichobezoar: Unveiling the Complexity of a 14-Year-Old's Abdominal Pain

**DOI:** 10.1155/cris/3934625

**Published:** 2024-12-09

**Authors:** Aiza Iqbal, Fatima Faraz, Bazigha Bano, Alishba Atta, Ayesha Azeem, Faizan Shahzad, Nimrah Jabeen, Besher Shami

**Affiliations:** ^1^Department of Medicine, Holy Family Hospital, Rawalpindi, Pakistan; ^2^Department of Surgery, Holy Family Hospital, Rawalpindi, Pakistan; ^3^Department of Medicine, Aleppo University Hospital, Aleppo, Syria

**Keywords:** case report, surgery, trichobezoar, trichophagia

## Abstract

Trichobezoars are accumulations of undigested hair. Usually, this disorder follows a psychiatric etiology; however, sometimes a nonpsychiatric etiology, such as pica, can also be suspected. Rapunzel syndrome is a rare type of trichobezoar in which the hair is usually confined to the stomach and small intestine. The authors present a rare case of trichobezoar in a young female without any psychiatric symptoms. Trichobezoar results in nonspecific GI symptoms and this causes delays in its diagnosis. It should always be considered a differential in a young female with nonspecific GI symptoms, especially in those with evidence of iron deficiency anemia.

## 1. Introduction

Bezoars are accumulations of partially digested or undigested hair or vegetable matter, trichobezoar, and phytobezoar, respectively [1]. Trichobezoar forms not due to impaired gastrointestinal motility like phytobezoars but rather due to psychiatric disorders in which hair is ingested. Rapunzel syndrome is a rare type of trichobezoar, in which the trichobezoar is usually confined to only the stomach and extends beyond the pylorus into the small intestine. The term “Rapunzel” denotes the fairytale of Rapunzel in which the princess Rapunzel, entrapped in a tall tower, has very long hair which she lets down through the window for her mother to climb. Most cases of trichobezoars occur in females, probably due to their long hair [2].

Clinicians need to recognize trichobezoars early on because, in addition to the distressing signs and symptoms, trichobezoars could lead to serious complications later on. Previous research identified these complications, such as bowel obstruction, gangrene, gastric perforation bleeding per rectum, and pancreatitis [3]. One study also identified pelvic abscess as a complication of laparotomy performed for gastric trichobezoar [4].

## 2. Case Presentation

We present the case of a 14-year-old girl who presented to the Outpatient Department of Surgery with a complaint of vomiting with increasing frequency for 4 months and epigastric pain for 1 month but more severe for 3 days. Both were associated with food intake. The patient also had a history of weight loss (45–40 kg). The patient had no prior medical conditions or surgical interventions. She was taking no medications. Her mother revealed that the patient had trichophagia for the past year.

The patient has visited many GP clinics for 3 months before presentation in our tertiary care setting; however, many of the treatments were symptomatic and addressed only the abdominal pain and vomiting and only relieved the symptoms temporarily. When her symptoms became very severe and unable to be alleviated by analgesics and antiemetics, a local GP examined her and gave her the diagnosis of splenomegaly, and she was investigated along those lines, which further led to delay in the diagnosis of the patient.

On examination, she was a thin, lean young girl with vitals of blood pressure: 110/70 mm/Hg, pulse: 90 beats/min, respiratory rate: 16 breaths/min, temperature: afebrile, a puffy face and pallor of the conjunctiva. She had pitting edema in both her feet. Abdominal examination revealed a tense, tender abdomen with a mass felt in the epigastric and right upper quadrant reaching down to the umbilicus. There was a mild tenderness present in the epigastric region. Cardiovascular, respiratory, and CNS systems were intact.

The patient's hematologic profile showed significant abnormalities, including severe anemia with hemoglobin at 5.3 g/dL (normal: 12.0–16.0) and hematocrit at 18% (normal: 40%–50%). Red cell indices were also low, with MCV at 64 Fl, MCH at 18 Pg, and MCHC at 29 g/dL, indicating microcytic, hypochromic anemia. RDW-CV was elevated at 21% (normal: 11%–16%), and RBC (red blood cell) count was below the normal range at 2.87 million/cm^3^ (reference range: 4.5–6.0 million/cm^3^), suggesting a potential issue with RBC production or loss, which required further evaluation to determine underlying causes. Platelets were reduced at 105,000/cm^3^ (normal: 140,000–450,000).

The patient's WBC (white blood cell) count was within the normal range at 5900 cells/cm^3^ (reference range: 4000–11,000 cells/cm^3^), indicating no current infectious etiology.

The patient's sodium was normal at 136 mEq/L, but potassium was low at 2.8 mEq/L (normal: 3.5–5.0), indicating hypokalemia.

Other lab investigations like renal function tests, liver function tests, and hepatitis serology were normal.

**Figure 1:**
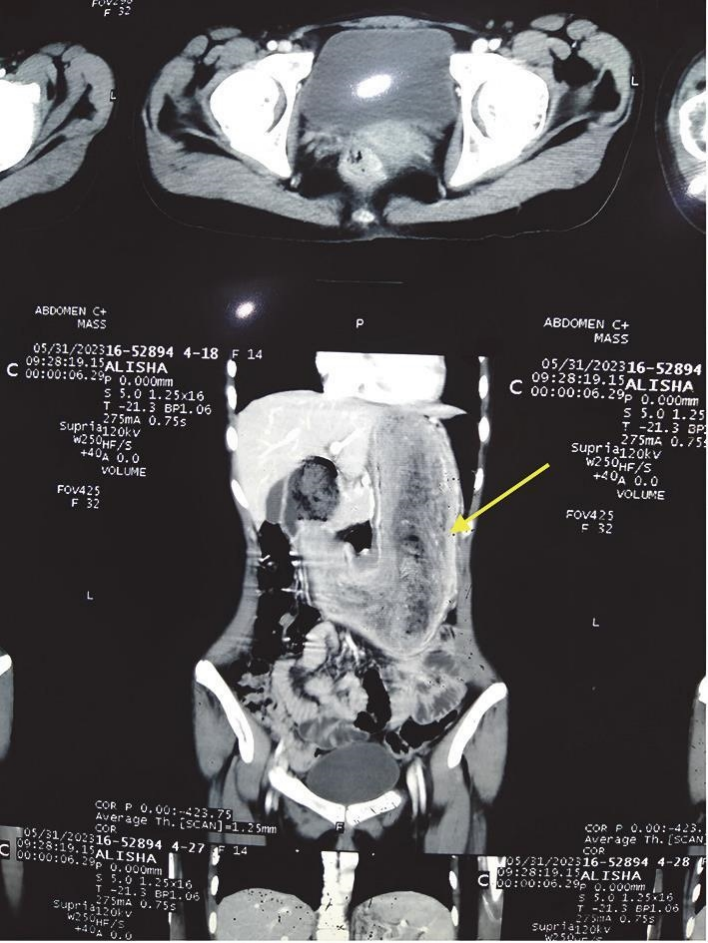
CT scan abdomen and pelvis of the patient. Large heterogeneous lesion (arrow) filling gastric lumen and duodenal bulb; incidental retroaortic left renal vein noted.

CT scan abdomen and pelvis was also ordered. The CT scan images are shown in Figure 1.

A contrast-enhanced CT scan revealed a large, well-defined, high-density lesion with internal mottled lucencies and varying densities, occupying nearly the entire gastric lumen and extending into the duodenal bulb. The remainder of the bowel and surrounding organs appeared normal. An incidental finding of a retroaortic left renal vein was also noted.

Upper GI endoscopy revealed a large trichobezoar, mixed with food particles, occupying the gastric lumen and extending into the duodenum up to the D3 segment, causing gastric outlet obstruction.

Regarding histopathology, since this institution is a public hospital in a resource-limited third-world country, the specimen for histopathology was not sent.

Our patient was initially referred by the GPE to the medical department with the diagnosis of splenomegaly. Coming from a lower-income background with limited awareness of the psychiatric aspects of trichophagia, she had not been assessed by a psychiatrist until she presented to the surgical department, where a detailed history and examination raised suspicion of trichophagia that further investigation began. A multidisciplinary team meeting, including surgeons, medical specialists, nutritionists, radiologists, and psychiatrists, was held, and imaging was ordered. The gastroenterology department was involved in an upper GI endoscopy, and exploratory laparotomy was subsequently performed, confirming the diagnosis of trichobezoar. The patient was then referred to the psychiatry department after discharge for management of trichophagia.

The patient was started on total parenteral nutrition, received three units of red cell concentrates (RCCs), and was scheduled for an exploratory laparotomy. Five days postadmission, the patient underwent the procedure. A midline incision was made, allowing visualization of the abdominal organs. An incision on the stomach confirmed the diagnosis of trichobezoar. A large trichobezoar, extending through nearly the entire stomach and the proximal part of the duodenum, was identified and successfully removed.

**Figure 2:**
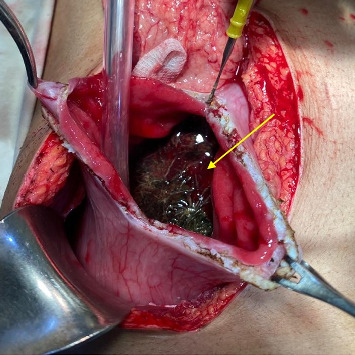
Intraoperative specimen of a large trichobezoar (arrow) extending through nearly the entire stomach and into the duodenum.

**Figure 3:**
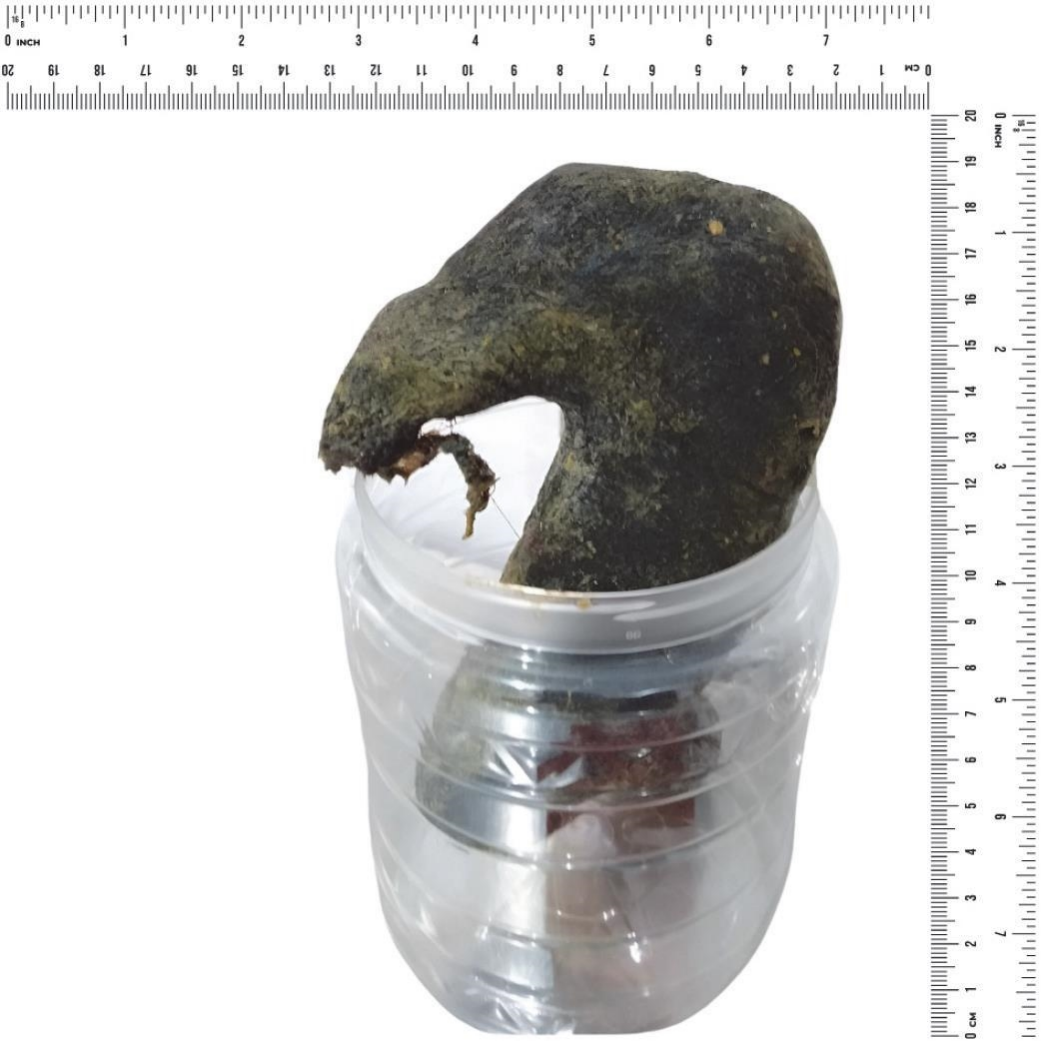
Gross specimen of the trichobezoar.

The images of intraoperative and gross specimens are shown in Figures 2 and 3.

Double-layer gastric repair was done, an abdominal drain was placed, and the abdomen was closed. Postoperatively, four FFPs were transfused, and the patient was kept NPO (Nil-Per-Os, meaning the patient could not intake any food or water by mouth).

The patient did not experience any postup complications related to laparotomy. Salt-free albumin was administered once a day for the next 3 days to combat the patient's hypoalbuminemia. Oral feeding was started on the 5th postoperative day, and the patient was discharged on the 6th postoperative day with a referral to the psychiatry department for counseling. Postoperative follow-up of the patient after 10 days revealed the patient to be healthy with no more gastrointestinal symptoms and recovering well.

## 3. Discussion

Gastric bezoars refer to the buildup of foreign materials, either undigested or partially digested, within the stomach [5]. Bezoars are classified based on the core substance that they are made up of—for example, we have lactobezoars—which are made up of milk proteins, phytobezoars—composed of plant fibers, trichobezoars-made up of hair, pharmacobezoars—made up of medications [6], and there also some miscellaneous types of bezoars noted in the literature, that is, metal bezoars [7], bezoars made up of dust plus nail particles (occupational exposure to dust + history of nail biting) [8], cement bezoars [9], plaster bezoars [10], and bezoars made up of paper or Styrofoam [11].

Human hair is indigestible due to its keratin composition and smooth surface, which resists peristalsis and gathers in the stomach. Over time, ingested hair accumulates into a large, shiny, black mass known as a trichobezoar, which can obstruct the gastric outlet [12, 13].

Trichobezoars are mostly present in young adolescent girls [10]. Two retrospective studies published regarding trichobezoars in pediatric populations spanning a period of 6 and 10 years revealed all the patients to be female [14, 15]. However, there also have been cases of trichobezoars in the male population [3, 16].

Trichobezoars are typically linked to trichotillomania (compulsive hair pulling) and trichophagia (hair ingestion). Trichotillomania affects about 1% of the population, with one-third also experiencing trichophagia, often in those with psychiatric disorders or intellectual disabilities [17–19].

Interestingly, our patient had no history of trichotillomania; instead, she ingested hair that fell in front of her face while still attached to her scalp. A thorough history revealed no psychiatric disorders or distressing life events. Previous research also indicates that not all cases of trichophagia-related trichobezoars involve psychiatric symptoms [20]. Iron deficiency anemia may have triggered her hair ingestion through pica. However, since trichobezoars can also cause iron deficiency due to poor nutrition, it's unclear which came first [12]. Possible explanations for anemia caused by trichobezoars include chronic blood loss from gastric mucosal ulceration, inflammation from mechanical irritation, malabsorption, gut obstruction, and reduced food intake due to early satiety and postmeal discomfort [2, 13].

Patients with Rapunzel syndrome are usually young females and may present with abdominal pain (most common), nausea and vomiting, symptoms of gastric outlet obstruction, anorexia, and weight loss [21], and even nutritional deficiencies like Iron deficiency anemia due to malabsorption. Patients can also present for the first time with complications of trichobezoar, like gastric erosion, ulceration, perforation, peritonitis [22], hematemesis, acute pancreatitis, obstructive jaundice, or intussusception. One study even reported trichobezoar as a cause of Mallory Weiss syndrome [23].

Delayed diagnosis is a major factor in trichobezoar complications. Trichobezoars may present as a nontender abdominal mass, often mistaken for fecal impaction, splenomegaly (as with our patient's initial exam by a general practitioner), or malignancy. Additionally, hair ingestion may go unreported unless specifically asked about [24, 25]. Diagnosis of Rapunzel syndrome is mainly through upper GI endoscopy. Various imaging techniques can be used like ultrasound, barium meal, CT scan, and MRI. CT scan has been described as superior to ultrasound and barium swallow because it allows clear visualization of the trichobezoar along with establishing the level of gastrointestinal obstruction [26].

One of the complications that occurred in our case was edema in the face and feet due to hypoalbuminemia. Possible reasons include poor intake, malabsorption, irritation of small bowel mucosa due to the tail of trichobezoar, or even lymphatic obstruction due to trichobezoar leading to increased protein loss [26–28].

Different treatment modalities can be used for the treatment of trichobezoar. These are chemical (Coca-Cola or Acetylcysteine), endoscopic, or surgical. Chemical dissolution with Coca-Cola combined with endoscopic extraction has been successful in the treatment of trichobezoar [28]. However, in Rapunzel syndrome, due to the tail extending into the duodenum, even a trial of endoscopic removal can cause the breaking off of parts of the trichobezoar, which cannot be retrieved endoscopically, so it should not be tried.

Surgical removal includes laparoscopic surgery and conventional open surgery [29]. Laparoscopic surgery is minimally invasive and is associated with good cosmetic results, early discharge, and a low rate of postoperative complications. Despite all these methods, laparotomy is still considered the treatment of choice for large trichobezoars, especially in cases of Rapunzel syndrome [30], since it has higher success rates, low rates of complications, and the prospect of exploring the whole gastrointestinal tract if needed [31]. The risks of complications associated with endoscopy and laparoscopy cannot outweigh their minimal invasiveness [30, 32]. Robot-assisted removal of trichobezoar has also been performed [31].

In the case of trichobezoar, only surgical removal of the trichobezoar is not enough. Proper psychiatric referral and follow-up of the patient should be ensured (as trichotillomania and trichophagia are mostly associated with psychiatric disorders) to prevent the recurrence of the condition, which occurs in up to 20% of the patients [33].

## 4. Conclusion

Trichobezoar presents with nonspecific signs and symptoms of epigastric pain, vomiting, weight loss, and a mass, often in young females. This often leads to delays in diagnosis and treatment, so trichobezoar should always be considered a differential diagnosis in young females presenting with these symptoms. Clinicians should particularly inquire about the risk factors associated with bezoar formation, like any psychiatric issues or previous gastric surgery; they should specially ask about the history of hair loss and look for signs of alopecia in patients presenting with these symptoms.

## Data Availability

Data are available upon request.
